# Systolic blood pressure lower than 130 mmHg in heart failure with preserved ejection fraction: a systematic review and meta-analysis of clinical outcomes

**DOI:** 10.1038/s41440-025-02240-w

**Published:** 2025-05-23

**Authors:** Chisa Matsumoto, Michiaki Nagai, Keisuke Shinohara, Nagisa Morikawa, Hisashi Kai, Hisatomi Arima

**Affiliations:** 1 Center for Health Surveillance & Preventive Medicine, Tokyo Medical University Hospital, Tokyo, Japan; 2 Department of Cardiology, Tokyo Medical University, Tokyo, Japan; 3 Cardiovascular Section, Department of Medicine, University of Oklahoma Health Science Center, Oklahoma City, OK USA; 4 Department of Cardiology, Hiroshima City Asa Hospital, Hiroshima, Japan; 5 Department of Cardiovascular Medicine, Faculty of Medical Sciences, Kyushu University, Fukuoka, Japan; 6 Division of Cardio-Vascular Medicine, Department of Internal Medicine, Kurume University School of Medicine, Fukuoka, Japan; 7 Department of Community Medicine, Kurume University School of Medicine, Fukuoka, Japan; 8 Department of Cardiology, Kurume University Medical Center, Kurume, Japan; 9 Department of Preventive Medicine and Public Health, Fukuoka University, Fukuoka, Japan

**Keywords:** HFpEF, optimal blood pressure control, meta-analysis, JSH2025

## Abstract

The optimal blood pressure (BP) management level for patients with heart failure (HF) with preserved ejection fraction (HFpEF) remains unclear. In conjunction with the upcoming the Japanese Society of Hypertension Guidelines for the Management of Hypertension 2025 (JSH2025), we conducted a systematic review and meta-analysis to evaluate whether managing systolic BP (SBP) < 130 mmHg improves outcomes in HFpEF patients. We searched PubMed, Cochrane and Ichishi for randomized controlled trials (RCTs) published since 2012 that targeted HFpEF patients; used strict BP control, antihypertensive medications, or intensive HF management as interventions; demonstrated significant BP reduction with achieved SBP < 130 mmHg in intervention groups; and had follow-up periods ≥6 months. Six studies were included, evaluating mineralocorticoid receptor antagonists (*n* = 2), angiotensin receptor-neprilysin inhibitors (*n* = 2), intensive BP control (*n* = 1), and intensive HF management (*n* = 1). Meta-analysis showed that achieving SBP < 130 mmHg significantly reduced HF hospitalizations (relative risk [RR] [95% confidence interval (CI)] 0.80 [0.69–0.93], *p* = 0.005) and demonstrated a trend toward reduced all-cause mortality (RR [95% CI] 0.74 [0.53–1.04], *p* = 0.083). While hypotension increased (RR [95% CI] 1.35 [1.03–1.79], *p* = 0.03), there was no significant increase in renal dysfunction or serious adverse events. Despite limitations from indirectness (no RCTs specifically targeted SBP < 130 mmHg as primary intervention), our findings suggest that achieving SBP < 130 mmHg in HFpEF patients may improve clinical outcomes. We recommend managing HFpEF patients to achieve SBP < 130 mmHg, while carefully monitoring for hypotension.

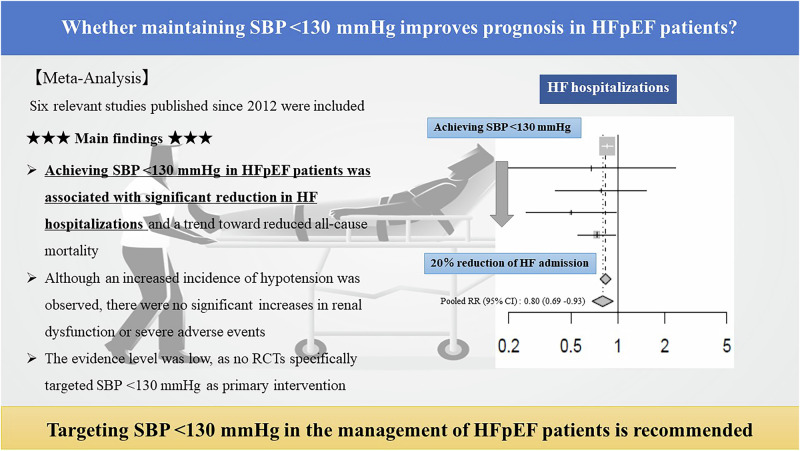

## Introduction

The global burden of heart failure (HF) is becoming an increasingly critical issue. According to statistical projections from 2017, an estimated 64 million people were affected by HF globally [1]. HF encompasses diverse pathophysiological conditions and is classified based on left ventricular ejection fraction (LVEF) into three categories: HF with reduced ejection fraction (HFrEF), HF with mildly reduced ejection fraction (HFmrEF), and HF with preserved ejection fraction (HFpEF). HFpEF accounts for approximately half of all HF cases, although the exact proportion varies depending on the definition used and geographical location [2]. Nonetheless, treatment strategies for HFpEF are not well established compared to those of HFrEF and the prognosis of HFpEF remains poor, presenting a significant challenge in contemporary cardiovascular medicine. Notably, 60–80% of HFpEF patients have comorbid hypertension [3–7], suggesting that left ventricular hypertrophy (LVH) and diastolic dysfunction may play central roles in its pathophysiology. However, evidence regarding optimal blood pressure (BP) management targets in HFpEF patients remains insufficient, highlighting a critical gap in current therapeutic strategies. The Japanese Society of Hypertension (JSH) conducted a systematic review (SR) on optimal BP targets in HFpEF patients during the development of the JSH Hypertension Guidelines 2019 (JSH2019) [8, 9]. Based on their findings, they recommended maintaining systolic blood pressure (SBP) below 130 mmHg in HFpEF patients [9]. However, since the publication of this SR, the therapeutic landscape for HF and hypertension has evolved significantly with the introduction of novel therapeutic agents, such as sodium-glucose cotransporter-2 (SGLT-2) inhibitors, non-steroidal mineralocorticoid receptor antagonists (MRA), and angiotensin receptor-neprilysin inhibitors (ARNI). Given these substantial changes in the treatment environment for HFpEF, we conducted an updated SR and meta-analysis to evaluate whether maintaining SBP < 130 mmHg improves prognosis in HFpEF patients, incorporating the latest available evidence, in conjunction with the upcoming the JSH Hypertension guideline 2025 (JSH2025).

## Methods

This SR and meta-analysis were performed in line with the Preferred Reporting Items for Systematic Review and Meta-Analyses (PRISMA) statement [10]. The protocol for this review and meta-analysis was prospectively developed and registered in the International Prospective Register of Systematic Reviews (PROSPERO; CRD42024532181). All included studies obtained approval from their respective institutional review boards or ethics committees, and all participants provided written informed consent. As this study is a meta-analysis of previously published data, institutional review board approval was not required.

### Literature search

**Fig. 1:**
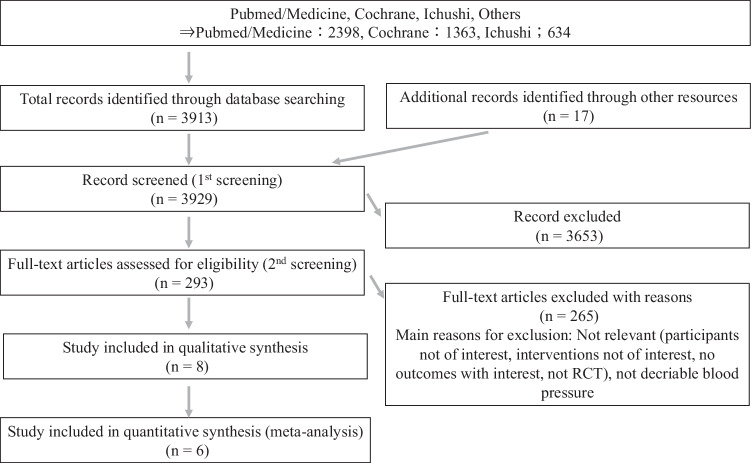
Flow chart of literature search of this study

We searched PubMed, Cochrane CENTRAL, and Igaku Chuo Zasshi (ICHUSHI: database of Japanese journals launched by the Japan Medical Abstracts Society) for studies published from January 2012 to May 2024 (Fig. 1). The selection of 2012 as the starting point was based on the European Society of Cardiology (ESC) guidelines published that year, which established a clear definition of HFpEF as HF with an LVEF greater than 50% [11]. Briefly, the literature search methodology involved using MeSH terms/keywords related to antihypertensive medications combined with MeSH terms/keywords for HFpEF. Animal studies were excluded, and the search was limited to publications in English and Japanese languages. The full literature search strategy is available in the Supplement Table 1. Two pairs of two independent reviewers (M.N., K.S., or C.M., N.M.) initially examined the titles and abstracts of all retrieved citations, followed by a thorough review of the full-text reports. Any disagreements were solved through consultation with another pair. Additionally, we performed forward and backward citation tracking of the included studies, and manually searched reference lists of relevant SRs. A reference manager software (Endnote, Clarivate, Version 21) was used to remove duplicates.

### Eligibility criteria

We included randomized controlled trials (RCTs) that met the following criteria: (1) RCTs targeting HFpEF patients with interventions involving intensive BP control, various antihypertensive medications, or strict HF management; (2) RCTs demonstrating significant BP reduction in the intervention group, with achieved SBP reaching <130 mmHg (including estimated values); and (3) RCTs with follow-up periods of 6 months or longer. Although only one study; the SPRINT follow-up study [12], explicitly set SBP < 120 mmHg as the intervention target, the intervention groups in all other included studies achieved SBP < 130 mmHg as a result of their interventions (drugs or intensive care). Therefore, regardless of the intervention method, we conducted the meta-analysis by defining the intervention group as achieved SBP < 130 mmHg. In cases where only a subgroup analysis population from a study met the inclusion criteria, inclusion of this subgroup alone was permitted [13]. Regarding the definition of HFpEF adopted in this study, we set it as LVEF > 40%, considering two factors: first, LVEF > 40% was widely used as the definition of HFpEF before the ESC established their definition in 2012 [11]; and second, even among RCTs published after 2012, majority of these trials were designed before 2012 when the ESC defined HFpEF as LVEF > 50% [11].

### Outcome measures

The outcomes of this meta-analysis were prespecified. The primary outcome of this meta-analysis was all-cause mortality and the secondary outcomes were cardiovascular (CVD) mortality (death due to cardiovascular disease and stroke), CVD (stroke, coronary artery disease, HF hospitalization, CVD mortality), HF hospitalization, renal dysfunction, hypotension, and serious adverse events (SAEs).

### Data extraction, assessment of risk of bias and indirectness

Two pairs of two independent reviewers (M.N., K.S., or C.M., N.M.) extracted data from eligible studies using a standardized data extraction form and checked independently by another pair. Any discrepancies were resolved by discussion by four independent reviewers (M.N., K.S., C.M., N.M.). The extracted data included study name; year of publication; study design; inclusion and exclusion criteria, contents of intervention, control group settings, baseline BP, BP after intervention, outcomes reported and outcome definitions. The risk of bias assessment was independently assessed by the same reviewers, using the criteria outlines in the Cochrane Risk-of-Bias tool for RCTs (RoB2) [14]. This tool includes the following domains: (1) Bias arising from the randomization process; (2) Bias due to deviations from intended interventions; (3) Bias due to missing outcome data; (4) Bias in measurement of the outcome; and (5) Bias in selection of the reported result. We evaluated indirectness for individual studies across the following domains: study population differences (applicability), intervention differences (applicability), difference in comparison groups, and outcome measurement differences [15]. Any disagreement between reviewers was resolved by discussion (M.N., K.S., C.M., N.M.).

### Statistical analysis

Both initial and achieved BP measurements across studies was summarized. When the achieved BP was not reported in the paper, we estimated it by subtracting the mean BP change from the mean baseline BP. In cases where only subgroup-specific mean BP changes (e.g., by sex) were reported rather than overall mean changes, we calculated the overall mean BP change using weighted averages and used it for the estimation [4, 16]. Overall baseline characteristics of patients included in the meta-analysis were summarized using appropriate descriptive statistics for both continuous and categorical variables. For continuous variables, data were expressed as weighted means. The weighted means were calculated by taking into account the sample size of each included study. For binary variables, we reported both the number of cases and proportions. When studies lacked data on specific variables’ means or counts, these studies were excluded from the calculation of the corresponding means or proportions. For outcomes, individual risk ratios (RRs) and their corresponding 95% confidence intervals (95% CI) for each study were calculated. A random-effects model using the restricted maximum-likelihood (REML) method was employed to generate pooled RR with 95% CI for data synthesis of meta-analysis. Heterogeneity across studies was assessed using the I² statistic according to the Cochrane Handbook guidelines [17]. The interpretation thresholds were as follows: I² values of 0% to 40% might not represent important heterogeneity; 30% to 60% may represent moderate heterogeneity; 50% to 90% may represent substantial heterogeneity; and 75% to 100% may represent considerable heterogeneity [17]. Potential publication bias was assessed using funnel plots generated with Cochrane’s Review Manager (RevMan 5.4) software, and Egger’s tests were performed if the number of included studies was greater than 10. For subgroup analyses, we conducted analyses stratified by intervention type (pharmacological interventions: MRA, ARNI, and non-pharmacological interventions: intensive HF care/strict BP control). We performed sensitivity analyses excluding post-RCT follow-up study: SPRINT [12] and study with substantial dropout rates during the run-in period: PARAGON-HF [4]. Furthermore, for the primary outcome of all-cause mortality, we conducted random-effects meta-regression analyses based on baseline SBP values, SBP in the intervention group, difference in achieved SBP between intervention and control groups, and changes in SBP from baseline in the intervention group. The EZR (Saitama Medical Center, Jichi Medical University), which is a graphical user interface for R (The R Foundation for Statistical Computing, version 2.13.0, Vienna, Austria) and SPSS 28 (IBM Corp., Armonk, NY) were used to conduct the meta-analyses.

### GRADE assessment

To evaluate the certainty of the evidence, we implemented the GRADE (Grading of Recommendations, Assessment, Development and Evaluations) approach [18, 19]. Based on this approach, we categorized evidence into four levels of certainty: high, moderate, low, and very low. The assessment process involved two pairs of two independent reviewers (M.N., K.S., or C.M., N.M.). In accordance with GRADE, RCTs were initially assigned high certainty ratings, which could subsequently be modified based on several key criteria. These criteria encompassed methodological quality (evaluated using the RoB2) [14], consistency across studies (particularly examining unexplained heterogeneity when I² exceeded 50% with *p* < 0.10), indirectness of evidence, precision of effect estimates (specifically examining whether confidence intervals crossed the threshold for minimal important differences), and potential publication bias [15]. The framework also permitted upgrading of evidence quality when warranted. Any disagreement between reviewers was resolved by discussion (M.N., K.S., C.M., N.M.).

## Results

### Study selection and patient characteristics

Our literature search identified 3929 studies. After screening titles and abstracts, 293 articles were selected for full-text evaluation. Finally, 6 studies were included in the meta-analysis (Fig. 1, Table 1). These included two studies investigating the efficacy of MRA [13, 20], two studies examining the utility of ARNI [4, 21], one follow-up study from the SPRINT study evaluating intensive BP control [12], and one study assessing the effectiveness of intensive HF management [22]. For the TOPCAT study, since significant BP reduction with MRA was observed only in the resistant hypertension group, only this resistant hypertension subgroup was included in the meta-analysis [13]. Regarding the follow-up study of the SPRINT, although achieved BP values were not reported [12], it was included because the main SPRINT paper documented that the intervention group achieved SBP < 130 mmHg [23]. Additionally, for the SR, we included two additional papers: a meta-analysis from the previous JSH2019 guideline examining similar clinical questions [8] and a review article on BP management in HFpEF patients [24], bringing the total to 8 papers.

**Table 1:** Characteristics of included studies

Study	Population	Follow up period	Intervention	Control	Baseline SBP (mmHg)	SBP after intervention (mmHg)	Outcomes	Notes
ALDO-HF	HFpEF (EF > 50%) *n* = 422 (mean age 67 y)	11.6 months (95% CI, 11.4-11.8 months)	Spironolactone *n* = 213	Placebo *n* = 209	Intervention: 135 ± 18 Control: 135 ± 18	Intervention: 128 (126–130) Control: 137 (135–139)	All-cause mortality, CVD, renal dysfunction、SAEs	
SPRINT	Patients who developed ADHF during follow-up in the SPRINT trial (EF ≧ 45%) *n* = 69 (mean age 76 y)	Median: 3.28 years (range 3 days to 5.44 years)	Targeting SBP < 120 mmHg *n* = 30	Targeting SBP < 140 mmHg *n* = 46	143.5 ± 19.6		All-cause mortality, CVD mortality, CVD, HF admission	Quasi-RCT of the SPRINT Although blood pressure data was not explicitly reported, included it based on blood pressure values from the parent SPRINT trial
PARAMOUNT	HFpEF (EF > 45%) *n* = 301 (mean age 71 y)	36 weeks	ARNI *n* = 149	Valsartan *n* = 152	Intervention: 136 Control: 136	Intervention: 128.5 Control: 134.5	All-cause mortality, CVD, HF admission renal dysfunction, hypotension, SAEs	
TOPCAT	HFpEF (EF ≧ 45%) *n* = 1004 (mean age 69 y) A subgroup analysis of the TOPCAT trial focusing on participants with resistant hypertension	3.1 years	Spironolactone *n* = 505	Placebo *n* = 499	Intervention: 133.4 ± 12.6 Control: 135.0 ± 13.9	Intervention: 129.3 ± 15.1 Control: 133.4 ± 16.9 (12 months after randomization)	All-cause mortality, CVD mortality, CVD, HF admission	
STRONG-HF	A subgroup analysis of the STRONG-HF trial stratified by ejection fraction, focusing on patients with Acute Heart Failure (EF > 40%) *n* = 347	180 days	High-intensity care (a high-intensity care strategy with early up titration of BB, ACE-I)	Usual care: followed according to local practice	Intervention: 125.1 ± 11.09 Control: 124.1 ± 12.00	Intervention: 119.6 ± 13.04 Control: 126.6 ± 15.91	All-cause mortality, CVD mortality, CVD, HF admission, renal dysfunction, hypotension, SAEs	In the intervention arm, SBP was reduced to <120 mmHg
PARAGON-HF	HFpEF (≧45%) over 50 years old	35 months (IQR: 30-41)	ARNI *n* = 2839	Valsartan *n* = 2407	Women 131 ± 16, men:130 ± 15	Difference of change in SBP; women: −4.3 ( − 5.6 to −3.0), men:−4.6 ( − 5.9 to −3.3) The weighted average SBP in the ARNI group : 129.68	All-cause mortality, CVD mortality, HF admission, hypotension, renal dysfunction, SAEs	Approximately 20% of participants dropped out during the run-in period.

*ADHF* Acute Decompensated Heart Failure, *IQR* inter quartile range, *ARNI* Angiotensin Receptor-Neprilysin Inhibitor, *BB* beta blocker, *ACE-I* ACE inhibitor, *SBP* systolic blood pressure, *CVD* cardiovascular disease, *SAEs* serious adverse events

The total number of participants included in the meta-analysis was 6939, with a mean age of 71.5 years, and 3557 (51.3%) were female. The mean LVEF was 58.4%. At baseline, SBP values were the same between the intervention and control groups, with a mean value of 131.2 mmHg. Following the intervention period, the achieved BP levels were 128.6 mmHg in the intervention group and 133.9 mmHg in the control group. (Table 2).

**Table 2:** Patients‘ characteristic of included studies

	Total	Intervention	control
N	6939	3481	3458
Age year	71.5	71.5	71.6
LVEF, %	58.4	58.4	58.3
Follow up period, weeks	26.6	26.5	26.7
Baseline SBP, mmHg	131.5	131.2	131.2
SBP after intervention, mmHg	131.5	128.6	133.9
HR, bpm	70	70.1	69.9
BMI	30.2	30.1	30.2
Women (%)	3557 (51.3)	1794 (52.0)	1763 (51.6)
NYHA I/II (%)	4666 (78.6)	2305 (78.2)	2361 (80.9)
NYHA III/IV (%)	1281 (21.6)	634 (21.5)	647 (22.2)
DM (%)	2781 (40.1)	1392 (40.3)	1379 (40.3)
DL (%)	975 (68.4)	485 (67.5)	490 (69.2)
HT (%)	6257 (95.9)	3148 (96.2)	3109 (95.7)
RAS-I (%)	5934 (85.6)	2982 (86.4)	2952 (86.3)
BB (%)	5271 (75.9)	2642 (76.6)	2629 (76.9)
CCB (%)	966 (67.7)	474 (66.0)	492 (69.5)
Diuretics (%)	6421 (92.5)	3222 (93.4)	3199 (93.6)
MRA (%)	3342 (48.2)	2004 (58.0)	1338 (39.1)

*LVEF* left ventricular ejection fraction, *SBP* systolic blood pressure, *HR* heart rate, *BMI* body mass index, *NYHA* New York Heart Association functional classification, *DM* diabetes mellitus, *DL* dyslipidemia, *HT* hypertension, *RAS-I* Renin Angiotensin system (RAS) inhibitors, *BB* beta blocker, *CCB* calcium channel blocker, *MRA* Mineralocorticoid Receptor Antagonists

The following variables contained missing values as indicated below. Variables not listed did not have any missing values

Total: EF: 1351, Follow up period: 4865*, BMI: 1351, NYHA I/II: 1073, NYHA III/IV: 1073, DM:69, DL:5513, HT: 416, CCB: 5513

Intervention: Age: 30, EF:712, Follow up period*: 2437, baseline SBP: 30, SBP after intervention; 30, HR:30, BMI: 712, NYHA I/II: 535, NYHA III/IV: 535, DM:30, DL:2763, HT: 207, RAS-I: 30, BB: 30, CCB: 2763, Diuretics: 30, MRA: 30

Control: Age: 39, EF:708, Follow up period*: 2428, baseline SBP: 39, SBP after intervention; 39, HR:39, BMI: 708, NYHA I/II: 538, NYHA III/IV: 538, DM:39, DL:2750, HT: 209, RAS-I: 39, BB: 39, CCB: 2750, Diuretics: 39, MRA: 39

*For the follow-up period, we included only studies that reported mean values in our calculation of the overall mean. Studies that reported only median values were excluded from this calculation

### Risk of bias within studies

Quality assessment using RoB2 (Supplemental Table 2) revealed that the included trials generally demonstrated low to unclear risk of bias across all domains. Also, none of RoB2 raters were authors of the included RCTs.

### Meta-analysis

#### All-cause mortality

**Fig. 2:**
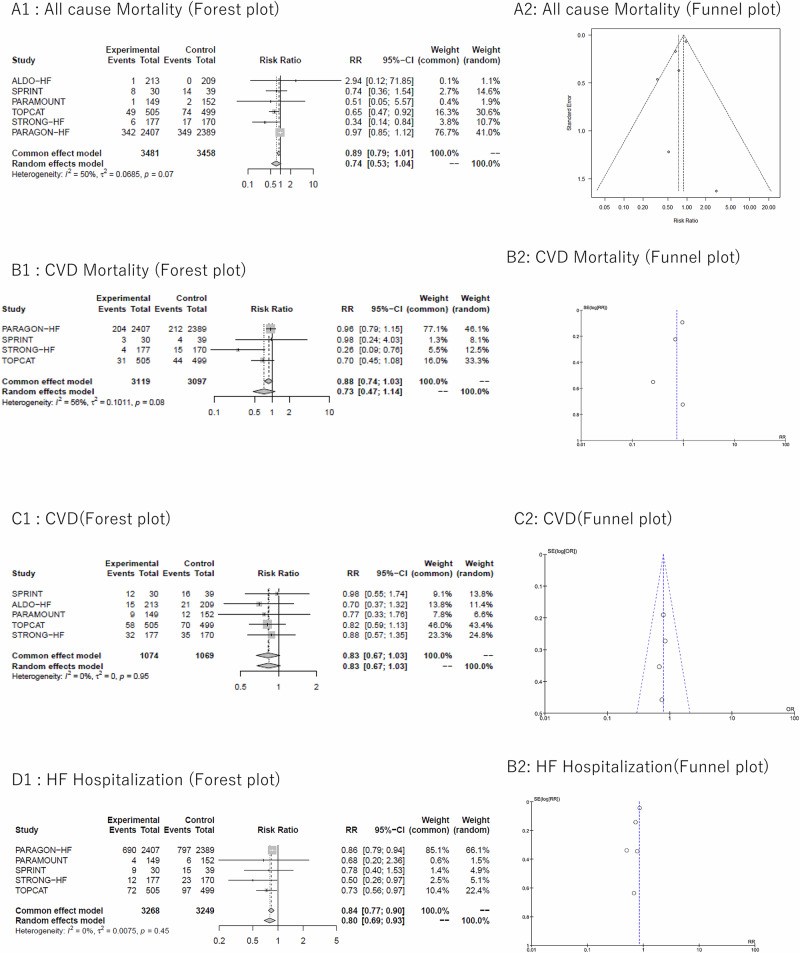

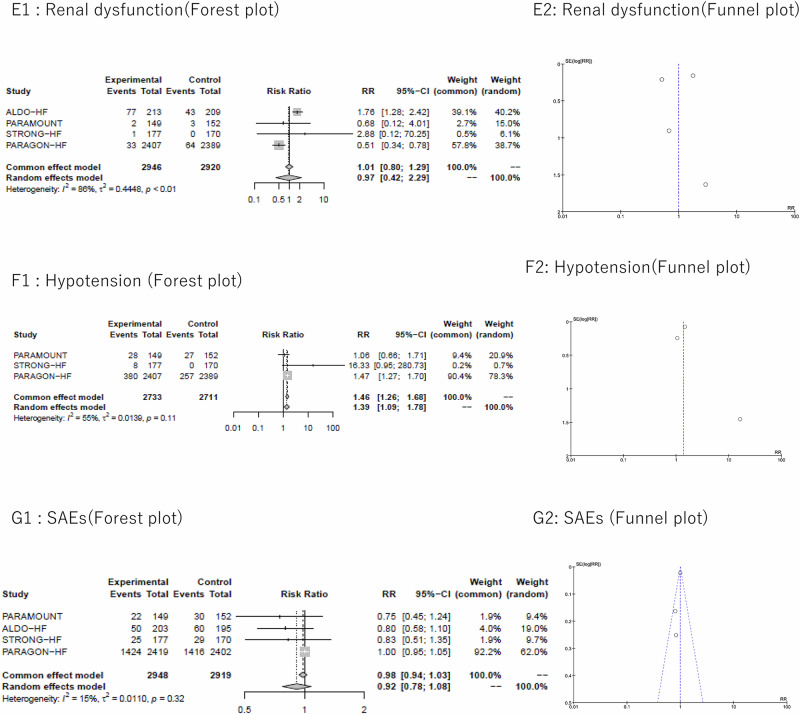
A1. Forest plot of all-cause mortality, Fig. 2 A2. Funnel plot of all-cause mortality. B1. Forest plot of CVD mortality, Fig. 2 B2. Funnel plot of CVD mortality. C1. Forest plot of CVD, Fig. 2 C2. Funnel plot of CVD. D1. Forest plot of HF Hospitalization, Fig. 2 D2. Funnel plot of HF Hospitalization. E1. Forest plot of renal dysfunction, Fig. 2 E2. Funnel plot of renal dysfunction. F1. Forest plot of hypotension, Fig. 2 F2. Funnel plot of hypotension. G1. Forest plot of SAEs, Fig. 2 G2. Funnel plot of SAEs

The meta-analysis for all-cause mortality included all six studies [4, 12, 13, 20–22]. In HFpEF patients, achieving SBP < 130 mmHg (intervention group) was associated with a 26% reduction in all-cause mortality compared to achieving SBP ≥ 130 mmHg (control group), showing a trend toward reduction although not statistically significant (RR [95% CI] 0.74 [0.53–1.04], *p* = 0.083). Moderate heterogeneity was observed (I² = 50%) (Fig. 2A). In sensitivity analyses, exclusion of the SPRINT [12], showed similar results (RR [95% CI] 0.72 [0.46–1.11], I² = 59%) (Supplemental Fig. 1A). On the other hand, analysis excluding the PARAGON-HF [4] showed significant mortality reduction with decrease of I² (RR [95% CI] 0.63 [0.47–0.84], I² = 0%) (Supplemental Fig. 1B).

In subgroup analyses by antihypertensive drug class and intervention method, the test for subgroup differences (random effects) yielded *P* = 0.05, indicating borderline significant heterogeneity between subgroups. MRA intervention, including the ALDO-HF [20] and the TOPCAT resistant hypertension sub-analysis [13], showed significant mortality reduction with RR (95% CI) of 0.67 (0.47-0.93) with decrease in I²(I² = 0%) (Supplemental Fig. 2).

**Fig. 3:**
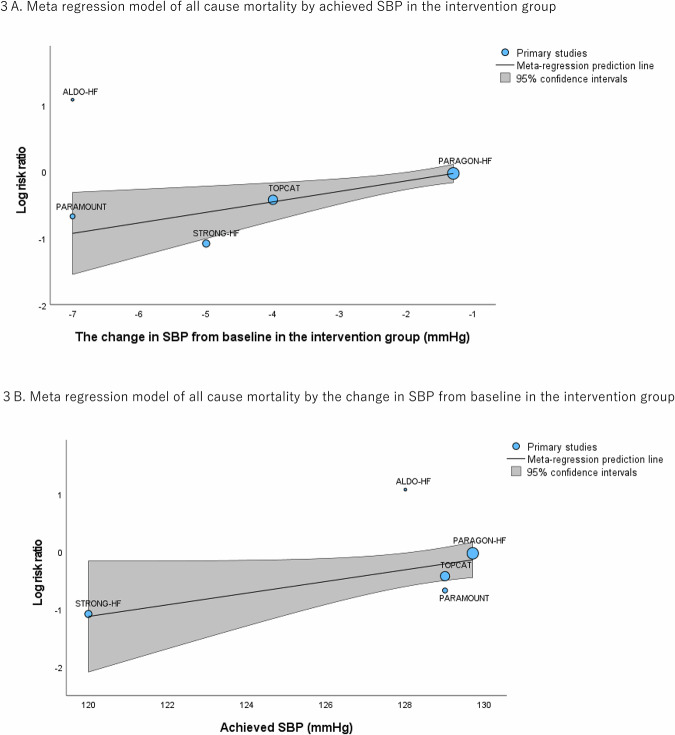
Meta regression model of all-cause mortality by achieved SBP in the intervention group and the change in SBP from baseline in the intervention group

In the meta-regression analysis, no significant associations were observed between all-cause mortality reduction and any of the following parameters: baseline SBP values (coefficient, 0.0185 [95% CI, −0.0916 to 0.129], *p* = 0.742) (Supplemental Fig. 3) or difference in achieved SBP between intervention and control (coefficient, 0.0995 [95% CI, −0.187 to 0.386], *p* = 0.497). On the other hand, significant associations were observed between all-cause mortality reduction and the following parameters of achieved SBP in the intervention group (coefficient, 0.117 [95% CI, 0.0238 to 0.211], *p* = 0.0140) and the change in SBP from baseline in the intervention group (coefficient, 0.158 [95% CI, 0.0425 to 0.273], *p* = 0.00733). (Fig. 3).

#### CVD mortality

Total 4 studies [4, 12, 13, 22] were included in a meta-analysis evaluating the effect of achieving SBP < 130 mmHg on CVD mortality. Achieving SBP < 130 mmHg in HFpEF had no significant impact on CVD mortality (RR [95% CI] 0.73 [0.47–1.14], *p* = 0.163). Moderate heterogeneity was observed (I² = 56%) (Fig. 2B). In subgroup analyses stratified by intervention type (antihypertensive medication [4, 13] or intensive care [12, 22]), the test for subgroup differences (random effects) was not statistically significant and achieving SBP < 130 mmHg did not demonstrate a significant reduction in CVD mortality in either intervention category. In sensitivity analyses, the exclusion of the SPRINT [12] did not alter these findings.

#### CVD Events

Five studies [12, 13, 20–22] were included in the meta-analysis examining CVD outcomes. Achieving SBP < 130 mmHg in HFpEF showed no significant effect on CVD events (RR [95% CI] 0.83 [0.67–1.03], *p* = 0.098). Low heterogeneity was observed (I² = 0%) (Fig. 2C). Subgroup analyses stratified by intervention type (antihypertensive medication [13, 20, 21] or intensive care [12, 22]) yielded similar results.

#### HF hospitalization

Five studies [4, 12, 13, 21, 22] were incorporated into the meta-analysis of HF hospitalization. In patients with HFpEF, achieving SBP < 130 mmHg was associated with a significant 20% reduction in HF hospitalization (RR [95% CI] 0.80 [0.69–0.93], *p* = 0.005). Heterogeneity was minimal (I² = 0%) (Fig. 2D). Similar to the all-cause mortality analysis, we conducted subgroup and sensitivity analyses, which showed that achieving SBP < 130 mmHg was associated with reduced HF hospitalization across all analyses without significant heterogeneity based on the test for subgroup differences (random effects).

#### Renal dysfunction

We included four studies [4, 20–22] in the meta-analysis examining renal dysfunction. Achieving SBP < 130 mmHg in HFpEF showed no significant impact on renal dysfunction (RR [95% CI] 0.97 [0.42–2.29], *p* = 0.953). Yet, high heterogeneity was observed (I² = 86%) (Fig. 2E). For subgroup analysis by intervention, the test for subgroup differences (random) was significant with *P* < 0.01. Subgroup analysis of ARNI intervention studies [4, 21] showed a significant 49% reduction in renal dysfunction with achieved SBP < 130 mmHg, with heterogeneity eliminated (RR [95% CI] 0.51 [0.34–0.77], *p* = 0.002; I² = 0%) (Supplemental Fig. 4).

#### Hypotension

Three studies were included in the meta-analysis of hypotension. [4, 21, 22] Achieving SBP < 130 mmHg in HFpEF increased hypotension by 39% (RR [95% CI] 1.39 [1.09-1.78], *p* = 0.007). Moderate heterogeneity was observed (I² = 55%) (Fig. 2F). For subgroup analysis by intervention, the test for subgroup differences (random effects) revealed borderline heterogeneity with *P* = 0.09. Subgroup analysis of ARNI intervention studies [4, 21] showed a significant 35% increase in hypotension (RR [95% CI] 1.35 [1.03–1.79], *p* = 0.03; I² = 39%) (Supplemental Fig. 5).

#### SAEs

Four studies [4, 20–22] were included in the meta-analysis examining SAEs. Achieving SBP < 130 mmHg in HFpEF did not increase SAEs (RR [95% CI] 0.92 [0.78-1.08], *p* = 0.295). Low heterogeneity was observed (I² = 15%) (Fig. 2G). Subgroup analyses by intervention method showed no increase in SAEs with no evidence of heterogeneity based on the test for subgroup differences (random effects). Of note, the incidence of SAEs was notably high in all studies, ranging from approximately 20% to 60% in both intervention and control groups [4, 20–22].

#### Indirectness and GRADE assessment

The certainty of evidence for maintaining SBP < 130 mmHg in HFpEF patients were evaluated by GRADE methodology. Regarding indirectness, there are several concerns in the intervention and population domains (Supplemental Table 2). For interventions, there is moderate/suspected indirectness since no pure RCTs specifically targeted achieving SBP < 130 mmHg as their primary intervention [4, 13, 20–22]. The quasi-RCT derived from the SPRINT that was included in this meta-analysis represents a follow-up study of a RCT of intensive BP control [12]. However, this study exhibits considerable indirectness as its study population was specifically restricted to participants who developed HF during the follow-up period. This post-hoc selection of participants based on an outcome that occurred after the original randomization may introduce significant selection bias. Also, several other factors contribute to indirectness in the study populations. For the sub-analysis of the TOPCAT, which investigated MRA effects in HFpEF, we included only patients with resistant hypertension [13]. Furthermore, the PARAGON-HF trial, examining ARNI effects, was limited to participants who could tolerate both valsartan and ARNI with approximately 20% dropout during the run-in period [4]. More importantly, except for ALDO-HF [20], five studies [4, 12, 13, 21, 22] defined HFpEF as LVEF ≥ 40-45%, meaning the meta-analysis population includes patients with HFmrEF, which further increases population indirectness.

Regarding outcome indirectness, among the three studies included in the hypotension meta-analysis, only the PARAMOUNT study explicitly specified “symptomatic hypotension. [21]” The other two studies used different definitions (“Hypotension” and “SBP < 100 mmHg”) for their event counts, which introduces indirectness [4, 22]. Also, for SAEs, we included “all-cause hospitalization” as a SAE in the ALDO-HF study [20], which diminishes directness. Across most outcomes, the level of indirectness was low to moderate. However, for renal dysfunction, a high degree of heterogeneity was observed (I² = 86%). Regarding publication bias, while funnel plot analysis did not visually suggest systematic bias, the assessment was limited by the small number of included studies (*n* = 6), precluding definitive evaluation. Based on these collective findings, the overall certainty of evidence in this meta-analysis was determined to be low, with particularly very low certainty for the outcome of renal dysfunction.

## Discussion

In HFpEF patients, BP management achieving SBP < 130 mmHg was associated with a significant reduction in HF hospitalizations and a trend toward lower all-cause mortality. Although an increased incidence of hypotension was observed, there were no significant increases in renal dysfunction or SAEs. However, the overall certainty of evidence was assessed as low, primarily due to indirectness, as SBP < 130 mmHg was not the targeted intervention in the majority of included studies. Nonetheless, considering the demonstrated benefits in critical outcomes - specifically HF hospitalizations and all-cause mortality - we strongly recommend targeting SBP < 130 mmHg in HFpEF patients, despite the limitations in evidence quality.

A previous meta-analysis conducted for the JSH2019, which examined the utility of SBP < 130 mmHg management in HFpEF patients, demonstrated similar benefits to our current study in reducing HF hospitalizations, but also showed an increase in renal dysfunction [8]. Both our meta-analysis and the JSH2019 meta-analysis consistently demonstrated that maintaining SBP below 130 mmHg effectively reduces HF hospitalizations. Indeed, considering the potential benefits of SBP < 130 mmHg management in reducing HF hospitalizations in HFpEF patients, major international hypertension guidelines, including ESH2023 [25] and ESC2024 [26], have incorporated recommendations for maintaining SBP < 130 mmHg in patients with HFpEF. The pathophysiological mechanism likely involves hypertension-induced LVH leading to diastolic dysfunction, as hypertension is one of predominant characteristics of HFpEF patients. Therefore, the beneficial effect of maintaining SBP below 130 mmHg on reducing HF hospitalizations may be partially attributed to the improvement in LVH and diastolic function through optimal BP control. Additional mechanisms underlying this benefit include the amelioration of coronary microvascular dysfunction, which may reduce myocardial ischemia and subsequent fibrosis development. BP control also plays a crucial role in preserving left atrial function by preventing atrial enlargement and maintaining its reservoir and contractile capabilities, thereby reducing the risk of pulmonary congestion and atrial fibrillation [24, 27]. Furthermore, appropriate BP management enhances endothelial function, which contributes to improved vascular compliance and reduced cardiac afterload through better vasodilatory responses and decreased inflammatory processes [24, 27].

Regarding hypotension, while the JSH2019 meta-analysis showed no significant increase with intervention [8], our study demonstrated an intervention-associated increase. However, it is noteworthy that among the five RCTs included in the JSH2019 meta-analysis for hypotension assessment [5–7, 28, 29], only one explicitly documented achievement of mean SBP < 130 mmHg in the intervention group [29], while the remaining studies did not reach this target. In contrast, all three RCTs incorporated in our meta-analysis achieved SBP < 130 mmHg with intervention. Moreover, only one of these studies included symptomatic hypotension as an outcome, and importantly, we found no increase in SAEs associated with intervention.

Therefore, while careful attention to hypotension is warranted in managing HFpEF patients with a target SBP < 130 mmHg, the demonstrated benefits in HF prevention suggest that optimal BP management remains highly valuable in this population.

For the impact of interventions on renal dysfunction, notable differences were observed between the JSH2019 meta-analysis [8] and our current analysis. The JSH2019 meta-analysis demonstrated a significant intervention effect with a RR (95% CI) of 1.52 (1.31–1.76) and minimal heterogeneity (I² = 0%) [8]. In contrast, our meta-analysis showed no significant intervention effect and exhibited substantially high heterogeneity. This discrepancy is particularly evident in two high-weight studies within our meta-analysis – the ALDO-HF [20] and the PARAGON-HF [4] - which showed opposing intervention effects: the ALDO-HF demonstrated an increased risk (RR: 1.76, 95% CI: 1.28–2.42) [20], while the PARAGON-HF showed a protective effect (RR: 0.51, 95% CI: 0.34–0.78) [4]. Regarding the renal effects of ARNI, the intervention used in PARAGON-HF [4], a pooled analysis of both PARADIGM-HF [30] (RCT investigating the prognostic benefits of ARNI in patients with HFrEF) and PARAGON-HF [4] revealed that the ARNI group, compared to the control group receiving renin angiotensin system (RAS) inhibitors, significantly attenuated the temporal deterioration of eGFR, particularly in patients with chronic kidney disease (CKD) stage 3, and reduced composite renal events by 44% [31]. Furthermore, a meta-analysis of RCTs in both patients with and without HF demonstrated that the improvement in renal outcomes with ARNI was significantly more pronounced in the HFpEF group compared to the HFrEF group with RR (95%CI) of 0.60 (0.44–0.82, I^2^ = 15%) [32]. The renoprotective mechanisms of ARNI can be explained by the complementary actions of its components: Valsartan and neprilysin inhibitor. Valsartan, the RAS inhibitor component, reduces intraglomerular pressure through efferent arteriolar vasodilation, which tends to decrease GFR. Conversely, neprilysin inhibition elevates natriuretic peptide (NP) levels, which increases renal perfusion through afferent arteriolar vasodilation, thereby enhancing GFR [33, 34]. NPs may also directly influence glomerular function by inducing relaxation of mesangial cells and podocytes, which increases capillary surface area and effective glomerular filtration area, potentially contributing to GFR maintenance through increased glomerular filtration coefficient [33, 34]. Additional renoprotective mechanisms include the suppression of local renal oxidative stress, inflammation, and fibrosis, along with inhibition of sodium reabsorption and tubular protein reuptake [33, 34]. Furthermore, when ARNI contributes to improved HF status, the subsequent enhancement in renal arterial pressure may also play a role in its renoprotective effects through improved renal perfusion.

Regarding the renal effects of MRAs, both the ALDO-AF [20] and the main paper of the TOPCAT study (which used spironolactone as intervention but was not included in our meta-analysis as the treatment-resistant hypertension sub-analysis paper lacked renal event data) [3] reported deterioration in renal function and increased incidence of hyperkalemia with spironolactone.

In contrast, finerenone, a novel non-steroidal MRA available since 2019, demonstrates higher MR selectivity and fewer adverse effects compared to spironolactone. The FINEARTS-HF trial, which evaluated finerenone’s prognostic benefits in HFpEF patients (excluded from our meta-analysis due to unclear post-intervention BP value), showed that while finerenone significantly reduced cardiovascular death and HF hospitalizations compared to placebo, it did not increase renal events [35]. Furthermore, a meta-analysis of RCTs investigating finerenone in diabetic kidney disease demonstrated significant reductions in renal events (RR: 0.77, 95% CI: 0.67–0.87) [36]. These findings suggest that renal effects in HFpEF patients likely vary depending on the type of MRA used. Considering that the aforementioned meta-analysis showed finerenone’s antihypertensive effect was only approximately 3 mmHg [36], the renoprotective effects of newer MRAs may not solely be attributed to BP reduction but potentially to MRA-specific mechanisms.

Furthermore, several important distinctions exist between our meta-analyses and the meta-analysis of JSH2019 [8]. The JSH2019 analysis included studies from 1996 to 2017, with interventions primarily consisting of RAS inhibitors, MRA (spironolactone), or beta-blockers [8]. In contrast, our meta-analysis for JSH2025 focused exclusively on studies published after 2012, where participants already had high baseline utilization of standard therapies: RAS inhibitors (85.6%), beta-blockers (75.9%), diuretics (92.5%), and MRAs (48.2%). Consequently, given that these studies examined intervention effects specifically in patients with established tolerability to these pharmacological agents, the observed outcomes may not solely reflect the impact of BP reduction per se, but might also be attributed to the pleiotropic effects of these medications beyond their antihypertensive properties. In fact, the mean achieved SBP levels in the intervention groups were 128.6 mmHg, with a relatively modest difference of 5.3 mmHg compared to the control groups in our meta-analysis. On the other hand, meta-regression analysis revealed significant associations between all-cause mortality reduction and achieved SBP in the intervention group. Also, reduction of all-cause mortality was associated with the change in SBP from baseline in the intervention group. This suggests that in addition to the non-antihypertensive effects of each antihypertensive agent, the degree of BP reduction itself may contribute to the suppression of all-cause mortality. However, while meta-regression analyses are typically considered robust when including 15 or more studies [37], this meta-regression analysis incorporated only 5 studies, necessitating careful interpretation of the results. Nevertheless, secondary analyses from major HFpEF trials have provided important observational data regarding BP targets. Sub-analysis from the TOPCAT demonstrated that a longer time in target range (TTR) for SBP between 110 and 130 mmHg was associated with reduced CVD risk in HPpEF [38]. Moreover, a U-shaped relationship was observed between time-averaged cumulative BP and outcomes (all-cause mortality, CVD mortality, and HF hospitalization), with the lowest risk observed at SBP 120–129 mmHg and DBP 70-79 mmHg [39]. These findings were corroborated by the sub-analysis of the PARAGON-HF, which similarly identified SBP 120–129 mmHg as the range associated with the most favorable prognosis [40]. However, since these studies were not specifically designed to evaluate BP reduction as the primary intervention, further evidence is needed to definitively establish the benefits of BP management targeting SBP < 130 mmHg or DBP < 80 mmHg in patients with HFpEF.

### Strengths and limitations

Our analysis has several notable methodological strengths. We implemented a comprehensive and systematic search protocol to identify all studies meeting eligibility criteria. Additionally, we employed the GRADE methodology to evaluate the overall quality and reliability of the evidence. Our study has several important limitations to consider. First, our meta-analysis does not include RCTs that specifically used SBP < 130 mmHg management as a primary intervention. Instead, we utilized RCTs targeting HFpEF populations where intervention groups achieved SBP < 130 mmHg, which introduces issues of indirectness. Despite numerous hypertension guidelines recommending SBP < 130 mmHg management for HFpEF patients, no dedicated RCTs have yet directly investigated this specific target. HFpEF represents a heterogeneous condition with diverse pathophysiological mechanisms beyond hypertension, including obesity, diabetes mellitus, atrial fibrillation, coronary artery disease, and CKD. This heterogeneity suggests that optimal BP targets may vary depending on individual patient characteristics and underlying pathophysiology. Furthermore, antihypertensive medications exert cardio- and renoprotective effects beyond mere BP reduction, which vary by drug class. While these factors make it challenging to investigate optimal BP management in HFpEF through conventional RCTs, future research should focus on evaluating treatment strategies tailored to specific subgroups to better inform individualized management approaches. Second, a notable limitation is that most studies included in our meta-analysis defined HFpEF as LVEF ≥ 40–45%, which means our analysis potentially incorporates patients with HFmrEF. Acknowledging the inconsistency in HFpEF definitions before the ESC 2012 HF guidelines [11], we deliberately restricted our meta-analysis to RCTs published from 2012 onward. Nevertheless, many of the included RCTs used definitions that differed from current standards. However, it is worth noting that the mean LVEF across RCTs incorporated in our study was 58.4%, suggesting that the majority of included patients would satisfy the contemporary definition of HFpEF with LVEF > 50%. Third, our meta-analysis included only six RCTs, and many outcomes demonstrated moderate to high heterogeneity. Also, several studies carrying substantial weight in our analysis presented challenges regarding indirectness. Furthermore, subgroup analyses by intervention type were based on extremely limited numbers of RCTs, suggesting the need for accumulation of additional evidence. Fourth, it is noteworthy that the BP change in the intervention groups across the RCTs included in our meta-analysis was minimal, with mean values of 131.2 mmHg at baseline and 128.6 mmHg post-intervention. This marginal reduction underscores the possibility that the observed benefits may be attributed to factors beyond mere BP reduction, potentially including specific pharmacological effects of the interventions used. Fifth, our SR exclusively included studies using antihypertensive medications as interventions, but other agents such as SGLT-2 inhibitors and glucagon-like peptide-1 receptor (GLP-1R) agonists have demonstrated BP-lowering effects [41–43]. Notably, SGLT-2 inhibitors, which now have indications for HF treatment, have been reported to reduce SBP in HFpEF patients while demonstrating cardiovascular benefits (primarily through reduced HF hospitalizations) [41]. Given the rapidly evolving landscape of HF management, ongoing evaluation of optimal BP targets in HFpEF patients will be necessary in the context of evolving standard therapies.

## Conclusion

In conclusion, although our meta-analysis did not include RCTs specifically designed with SBP < 130 mmHg as a primary intervention target, our findings demonstrate that achieving SBP < 130 mmHg in HFpEF patients was associated with significant reduction in HF hospitalizations and a trend toward reduced all-cause mortality. Despite an increase in hypotension, there was no significant increase in renal dysfunction or SAEs. Based on these findings, we recommend targeting SBP < 130 mmHg in the management of HFpEF patients. However, given the heterogeneous nature of HFpEF, this approach should be implemented with careful consideration of individual patient characteristics and vigilant monitoring for potential adverse effects including hypotension. Future research should focus on developing personalized treatment strategies that consider both the antihypertensive and pleiotropic effects of various pharmacological agents in this complex patient population.

## Supplementary information

Supplementary Figures

Supplementary Table 1

Supplementary Table 2

Hypertension Research: Checklist for Style
