# Supplementary material for: Systolic blood pressure lower than 130 mmHg in heart failure with preserved ejection fraction: a systematic review and meta-analysis of clinical outcomes
Source: Hypertens Res. 2025 May 23;48(8):2138–51. doi: 10.1038/s41440-025-02240-w (PMC12321576; doi:10.1038/s41440-025-02240-w)

Supplemental Figure 1A. Sensitivity analysis : All cause Mortality (Forest plot)

Study	Experimental Events	Experimental Total	Control Events	Control Total	RR	95%-CI	Weight (common)	Weight (random)
**RCT = 1**								
ALDO-HF	1	213	0	209	2.94	[0.12; 71.85]	0.1%	1.1%
PARAMOUNT	1	149	2	152	0.51	[0.05; 5.57]	0.4%	1.9%
TOPCAT	49	505	74	499	0.65	[0.47; 0.92]	16.3%	30.6%
STRONG-HF	6	177	17	170	0.34	[0.14; 0.84]	3.8%	10.7%
PARAGON-HF	342	2407	349	2389	0.97	[0.85; 1.12]	76.7%	41.0%
**Common effect model**		3451		3419	**0.89**	**[0.79; 1.01]**	**97.3%**	**--**
**Random effects model**					**0.72**	**[0.46; 1.11]**	**--**	**85.4%**
Heterogeneity: $I^2 = 59\%$, $\tau^2 = 0.1112$, $p = 0.04$								
**RCT = 0**								
SPRINT	8	30	14	39	0.74	[0.36; 1.54]	2.7%	14.6%
**Common effect model**		**3481**		**3458**	**0.89**	**[0.79; 1.01]**	**100.0%**	**--**
**Random effects model**					**0.74**	**[0.53; 1.04]**	**--**	**100.0%**

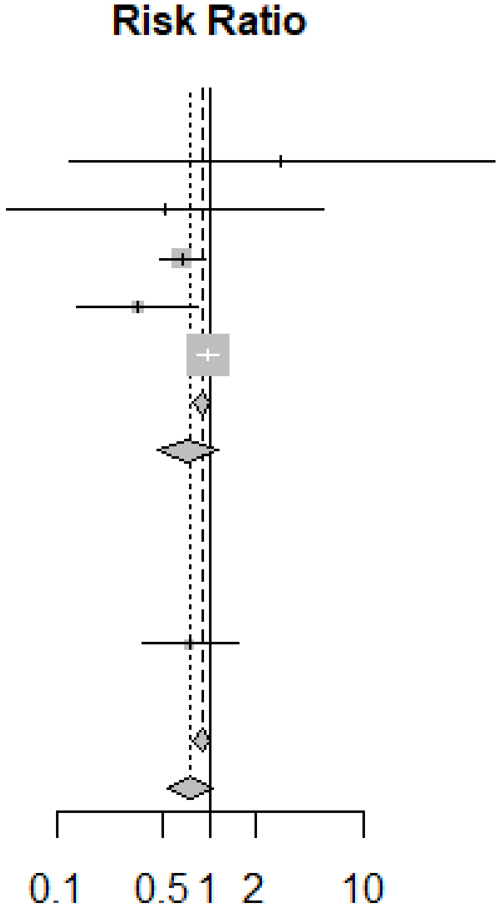

Heterogeneity: $I^2 = 50\%$, $\tau^2 = 0.0685$, $p = 0.07$
Test for subgroup differences (common effect): $\chi_1^2 = 0.24$, df = 1 ($p = 0.62$)
Test for subgroup differences (random effects): $\chi_1^2 = 0.01$, df = 1 ($p = 0.93$)

Supplemental Figure 1B. Sensitivity analysis : All cause Mortality (Forest plot)

Study	Experimental Events	Experimental Total	Control Events	Control Total	OR	95%-CI	Weight (common)	Weight (random)
ALDO-HF	1	213	0	209	2.96	[0.12; 73.02]	0.5%	1.1%
SPRINT	8	30	14	39	0.65	[0.23; 1.84]	9.4%	10.2%
PARAMOUNT	1	149	2	152	0.51	[0.05; 5.65]	2.1%	1.9%
TOPCAT	49	505	74	499	0.62	[0.42; 0.91]	70.5%	74.7%
STRONG-HF	6	177	17	170	0.32	[0.12; 0.82]	17.6%	12.1%
**Common effect model**		**1074**		**1069**	**0.58**	**[0.42; 0.80]**	**100.0%**	**--**
**Random effects model**					**0.58**	**[0.42; 0.81]**	**--**	**100.0%**

Heterogeneity: $I^2 = 0\%$, $\tau^2 = 0$, $p = 0.61$

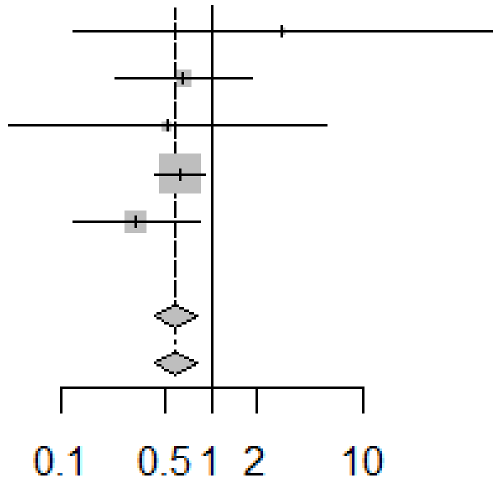

Supplemental Figure 2. Subgroup analysis by intervention : All cause Mortality (Forest plot)

Subgroup	Study	Experimental Events	Experimental Total	Control Events	Control Total	RR	95%-CI	Weight (common)	Weight (random)
MRA	Non.Specific.0.MRA1.ARNI2 = 1								
	ALDO-HF	1	213	0	209	2.94	[0.12; 71.85]	0.1%	1.1%
	TOPCAT	49	505	74	499	0.65	[0.47; 0.92]	16.3%	30.6%
	Common effect model		718		708	0.67	[0.48; 0.94]	16.4%	--
	Random effects model					0.67	[0.47; 0.93]	--	31.7%
	Heterogeneity: $I^2 = 0\%$, $\tau^2 = 0$, $p = 0.36$								
Intensive care	Non.Specific.0.MRA1.ARNI2 = 0								
	SPRINT	8	30	14	39	0.74	[0.36; 1.54]	2.7%	14.6%
	STRONG-HF	6	177	17	170	0.34	[0.14; 0.84]	3.8%	10.7%
	Common effect model		207		209	0.51	[0.29; 0.89]	6.5%	--
	Random effects model					0.53	[0.25; 1.13]	--	25.3%
	Heterogeneity: $I^2 = 43\%$, $\tau^2 = 0.1321$, $p = 0.19$								
ARNI	Non.Specific.0.MRA1.ARNI2 = 2								
	PARAMOUNT	1	149	2	152	0.51	[0.05; 5.57]	0.4%	1.9%
	PARAGON-HF	342	2407	349	2389	0.97	[0.85; 1.12]	76.7%	41.0%
	Common effect model		2556		2541	0.97	[0.85; 1.11]	77.1%	--
	Random effects model					0.97	[0.85; 1.11]	--	43.0%
	Heterogeneity: $I^2 = 0\%$, $\tau^2 = 0$, $p = 0.60$								
	**Common effect model**		**3481**		**3458**	**0.89**	**[0.79; 1.01]**	**100.0%**	**--**
	**Random effects model**					**0.74**	**[0.53; 1.04]**	**--**	**100.0%**

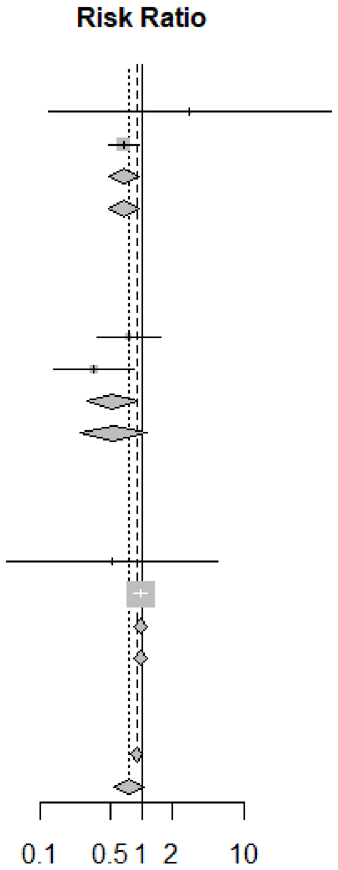

Heterogeneity: $I^2 = 50\%$, $\tau^2 = 0.0685$, $p = 0.07$
Test for subgroup differences (common effect): $\chi^2_2 = 8.06$, df = 2 ($p = 0.02$)
Test for subgroup differences (random effects): $\chi^2_2 = 6.12$, df = 2 ($p = 0.05$)

Supplemental Figure 3. Meta regression model of all cause mortality by baseline SBP in intervention group

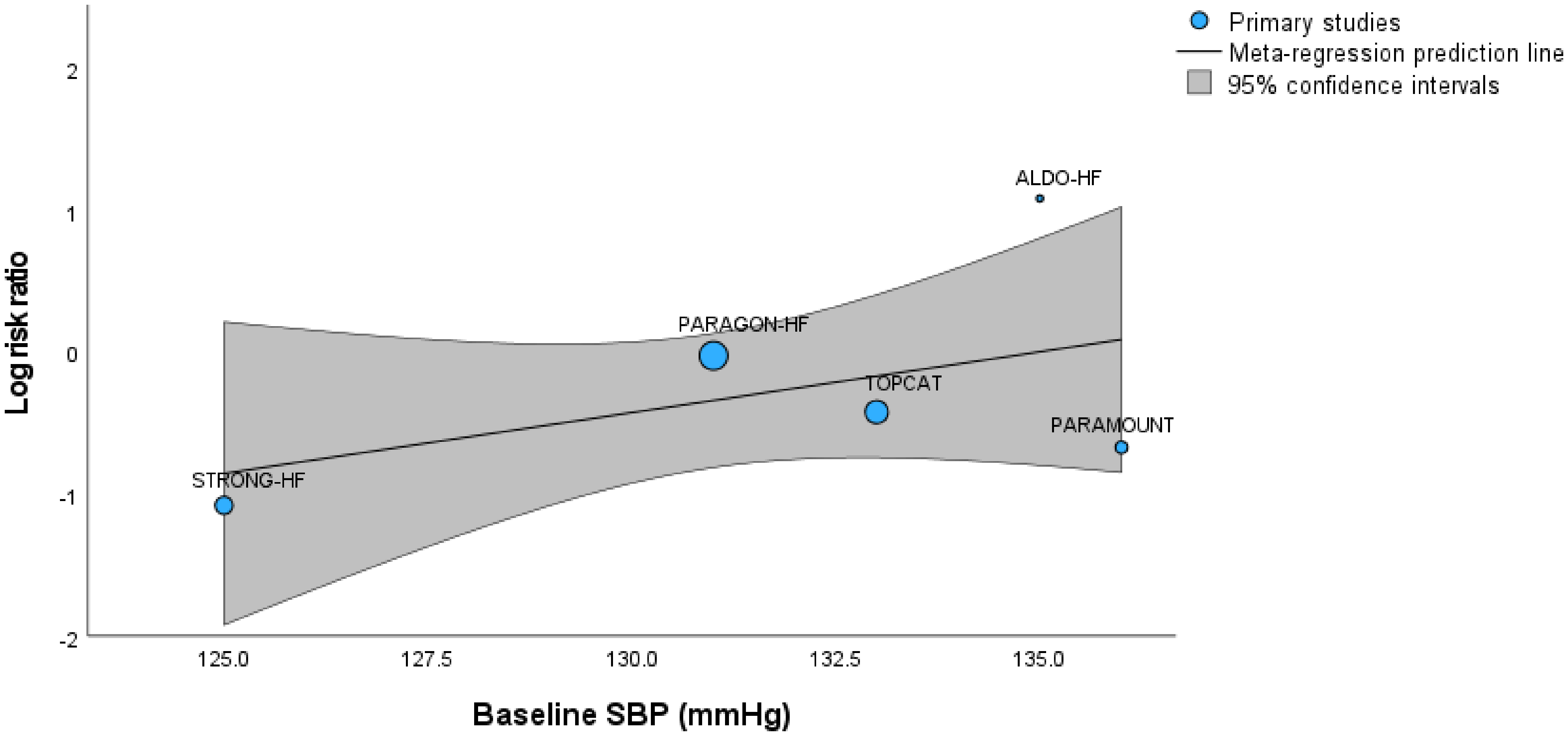

Supplemental Figure 4. Subgroup analysis by intervention : Renal dysfunction (Forest plot)

Subgroup	Study	Experimental Events	Experimental Total	Control Events	Control Total	OR	95%-CI	Weight (common)	Weight (random)
	Non.Specific.0.MRA1.ARNI2 = 1								
**MRA**	ALDO-HF	77	213	43	209	2.19	[1.41; 3.38]	29.3%	37.8%
	Non.Specific.0.MRA1.ARNI2 = 2								
**ARNI**	PARAMOUNT	2	149	3	152	0.68	[0.11; 4.10]	3.1%	16.9%
	PARAGON-HF	33	2407	64	2389	0.50	[0.33; 0.77]	67.0%	37.9%
	Common effect model		2556		2541	0.51	[0.34; 0.77]	70.1%	--
	Random effects model					0.51	[0.34; 0.77]	--	54.8%
	Heterogeneity: $I^2 = 0\%$, $\tau^2 = 0$, $p = 0.76$								
	Non.Specific.0.MRA1.ARNI2 = 0								
**Intensive care**	STRONG-HF	1	177	0	170	2.90	[0.12; 71.63]	0.5%	7.4%
	**Common effect model**		**2946**		**2920**	**1.02**	**[0.76; 1.35]**	**100.0%**	**--**
	**Random effects model**					**1.05**	**[0.40; 2.76]**	**--**	**100.0%**

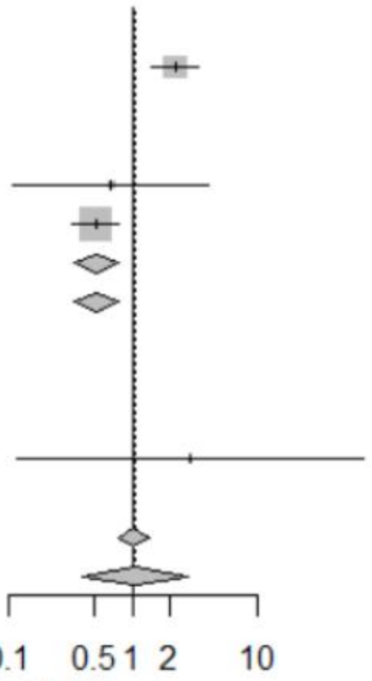

Heterogeneity: $I^2 = 87\%$, $\tau^2 = 0.5934$, $p < 0.01$
Test for subgroup differences (common effect): $\chi^2_2 = 22.80$, df = 2 ($p < 0.01$)
Test for subgroup differences (random effects): $\chi^2_2 = 22.78$, df = 2 ($p < 0.01$)

Supplemental Figure 5. Subgroup analysis by intervention : Hypotension (Forest plot)

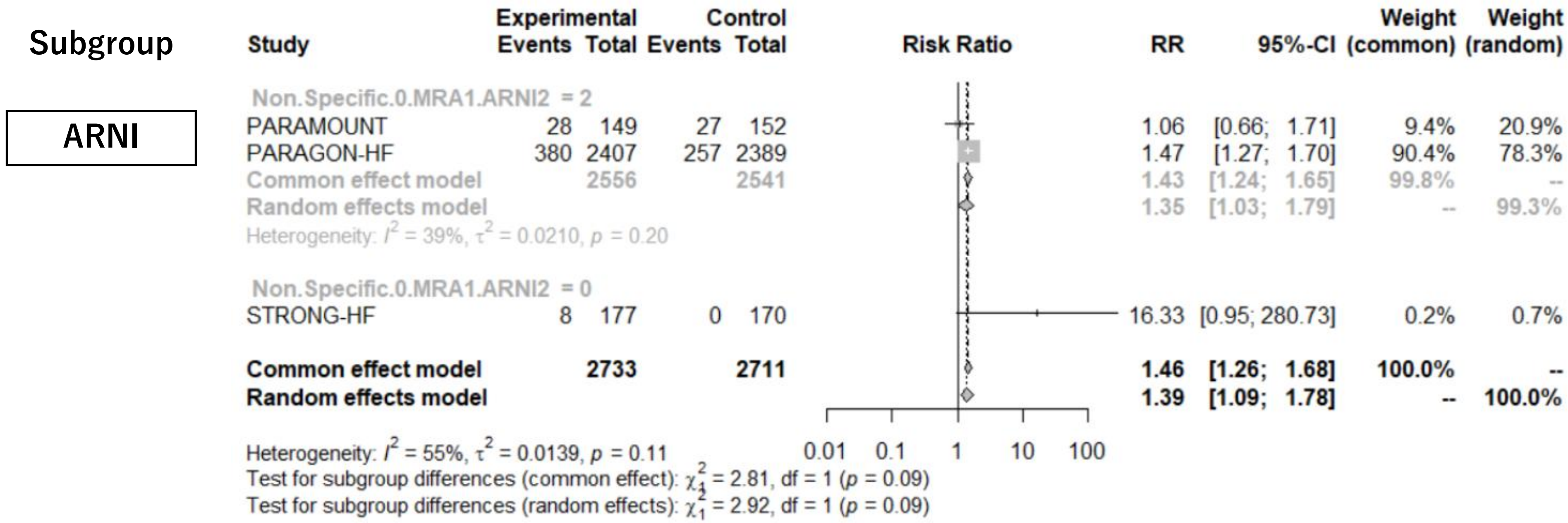

Supplement: Supplementary file 1 — Supplementary Figures [file 41440_2025_2240_MOESM1_ESM.pdf]
