# Supplementary material for: Systolic blood pressure lower than 130 mmHg in heart failure with preserved ejection fraction: a systematic review and meta-analysis of clinical outcomes
Source: Hypertens Res. 2025 May 23;48(8):2138–51. doi: 10.1038/s41440-025-02240-w (PMC12321576; doi:10.1038/s41440-025-02240-w)
Supplement: Supplementary file 2 — Supplementary Table 1 [file 41440_2025_2240_MOESM2_ESM.docx]

Supplement Table 1 : Literature search strategy

| Intervention/ Population | Pubmed/Medline: MeSH | Cochrane: Mesh | Title＆Abstract research words | Ichushi : Topic heading (TH) | Ichushi :Title＆Abstract research words |  |
| --- | --- | --- | --- | --- | --- | --- |
| Antihypertensive drug | Antihypertensive Agents[MeSH Terms] | Antihypertensive Agents | antihypertensive[Title/Abstract] | 降圧剤/TH | 降圧剤 |  |
|  | Hypertension/drug therapy[MeSH Terms] | Drug Therapy |  | 降圧作用/TH | 薬物療法 |  |
|  | Blood Pressure/drug effects[MeSH Terms] |  |  | 薬物療法/TH |  |  |
|  | Antihypertensive Agents/therapeutic use[MeSH Terms] |  |  |  |  |  |
|  | Antihypertensive Agents/ adverse effects [MeSH Terms] |  |  |  |  |  |
|  | Drug Therapy [MeSH Terms] |  |  |  |  |  |
|  | Pubmed/Medline: MeSH | Cochrane: Mesh | Title＆Abstract research words | Ichushi : Topic heading (TH) | Ichushi :Title＆Abstract research words |  |
| Blood pressure lowering |  |  | blood pressure target[Title/Abstract] |  | 厳格降圧 |  |
|  |  |  | BP target[Title/Abstract] |  | 降圧目標 |  |
|  |  |  | blood pressure goal[Title/Abstract] |  | 血圧管理 |  |
|  |  |  | BP goal[Title/Abstract] |  | 至適血圧 |  |
|  |  |  | intensive blood pressure[Title/Abstract] |  | 至適な血圧 |  |
|  |  |  | intensive antihypertensive[Title/Abstract] |  | "blood presssure control" |  |
|  |  |  | blood pressure lowering[Title/Abstract] |  | "tight blood pressure" |  |
|  |  |  | active blood pressure treatment[Title/Abstract] |  | "target blood pressure" |  |
|  |  |  | active antihypertensive treatment[Title/Abstract] |  | 厳格な降圧 |  |
|  |  |  | tight blood pressure[Title/Abstract] |  | "optimal blood pressure" |  |
|  |  |  | strict blood pressure[Title/Abstract] |  | "strict blood pressure |  |
|  |  |  | "blood pressure control"[Title/Abstract] |  |  |  |
|  |  |  | "goal blood pressure"[Title/Abstract] |  |  |  |
|  | MeSH 検索ワード |  | Title＆Abstract research words | Ichushi : Topic heading (TH) | Ichushi :Title＆Abstract research words |  |
| Heart Failure | Heart Failure[MeSH Terms] |  | heart failure with mildly reduced ejection fraction[Title/Abstract] | 心不全 | 心不全 |  |
|  |  |  | heart failure with mid-range ejection fraction [Title/Abstract] |  | HFmrEF |  |
|  |  |  | heart failure[Title/Abstract] |  | HFnEF |  |
|  |  |  | heart failure with preserved ejection fraction[Title/Abstract] |  | 収縮能の保たれた心不全 |  |
|  |  |  | HFpEF[Title/Abstract] |  | LVEFの保たれた心不全 |  |
|  |  |  | HFmEF[Title/Abstract] |  | 拡張不全型心不全 |  |
|  |  |  | "diastolic heart failure"[Title/Abstract] |  | heart failure |  |
|  |  |  | "preserved systolic function"[Title/Abstract] |  | heart failure with preserved ejection fraction |  |
|  |  |  | "normal systolic function"[Title/Abstract] |  | heart failure with mid-range ejection fraction |  |
|  |  |  | "HF-pEF"[Title/Abstract] |  |  |  |
|  |  |  | HFnEF[Title/Abstract] |  |  |  |
|  |  |  | HFmrEF[Title/Abstract] |  |  |  |
| Study design | MeSH Pubmed/Medline: MeSH | Cochrane: Mesh | Title＆Abstract research words | Publication Type | Ichushi : Topic heading (TH) | Ichushi :Title＆Abstract research words |
| Study design | Clinical Studies as Topic[MeSH Terms] |  | clinical trial[Title/Abstract] | Clinical Study[Publication Type] | ランダム化比較試験 | RCT |
|  | Clinical Trials as Topic[MeSH Terms] |  | RCT[Title/Abstract] | Clinical Trial[Publication Type] | 臨床試験 | ランダム |
|  | Controlled Clinical Trials as Topic[MeSH Terms] |  | randomly[Title/Abstract] | Controlled Clinical Trial[Publication Type] |  | 無作為 |
|  | Randomized Controlled Trials as Topic[MeSH Terms] |  | random allocation[Title/Abstract] | Randomized Controlled Trial[Publication Type] |  |  |
|  |  |  | randomized controlled trial[Title/Abstract] |  |  |  |
|  |  |  | controlled clinical trial[Title/Abstract] |  |  |  |
|  |  |  | randomiz*[Title/Abstract] |  |  |  |
|  |  |  | randomis*[Title/Abstract] |  |  |  |
| Others | MeSH Pubmed/Medline: MeSH and others |  |  |  |  |  |
| Exclusion of animal studies | NOT (animals[MH] NOT humans[MH]) |  |  |  |  |  |
| Search period | January 2012 ~ |  |  |  |  |  |
| Language | (english[Language]) OR (Japanese[Language]) |  |  |  |  |  |
