# Supplementary material for: Systolic blood pressure lower than 130 mmHg in heart failure with preserved ejection fraction: a systematic review and meta-analysis of clinical outcomes
Source: Hypertens Res. 2025 May 23;48(8):2138–51. doi: 10.1038/s41440-025-02240-w (PMC12321576; doi:10.1038/s41440-025-02240-w)
Supplement: Supplementary file 3 — Supplementary Table 2 [file 41440_2025_2240_MOESM3_ESM.docx]

Supplemental Table 2: Risk of Bias and Indirectness of included studies (Rob2)

All-cause death

| STUDY | | Risk of Bias | | | | |  | Indirectness | | | |  |
| --- | --- | --- | --- | --- | --- | --- | --- | --- | --- | --- | --- | --- |
| Study name | Study design | Bias arising from the randomization process | Bias due to deviations from intended interventions | Bias due to missing outcome data | Bias in measurement of the outcome | Bias in selection of the reported result | Summary | Population | Intervention | Control | Outcome | Summary |
| ALDO-HF | RCT | -1 | 0 | 0 | 0 | 0 | 0 | 0 | -1 | -1 | 0 | -1 |
| SPRINT | quasi- RCT | -1 | -1 | 0 | 0 | 0 | -1 | -2 | 0 | 0 | 0 | -2 |
| PARAMOUNT | RCT | -1 | 0 | 0 | 0 | 0 | -1 | 0 | -1 | -1 | 0 | -1 |
| TOPCAT | RCT | 0 | 0 | 0 | 0 | 0 | 0 | -1 | -1 | -1 | 0 | -1 |
| STTRONG-HF | RCT | 0 | -1 | 0 | 0 | 0 | -1 | -1 | -1 | -1 | 0 | -1 |
| PARAGON-HF | RCT | -1 | 0 | 0 | 0 | 0 | -1 | -1 | -1 | -1 | 0 | -1 |

CVD mortality

| STUDY | | Risk of Bias | | | | |  | Indirectness | | | |  |
| --- | --- | --- | --- | --- | --- | --- | --- | --- | --- | --- | --- | --- |
| Study name | Study design | Bias arising from the randomization process | Bias due to deviations from intended interventions | Bias due to missing outcome data | Bias in measurement of the outcome | Bias in selection of the reported result | Summary | Population | Intervention | Control | Outcome | Summary |
| SPRINT | quasi- RCT | -1 | 0 | 0 | 0 | 0 | -1 | -2 | 0 | 0 | -1 | -2 |
| TOPCAT | RCT | 0 | 0 | 0 | 0 | 0 | 0 | -1 | -1 | -1 | 0 | -1 |
| STTRONG-HF | RCT | 0 | -1 | 0 | 0 | 0 | -1 | -1 | -1 | -1 | 0 | -1 |
| PARAGON-HF | RCT | -1 | 0 | 0 | 0 | 0 | -1 | -1 | -1 | -1 | 0 | -1 |

CVD

| STUDY | | Risk of Bias | | | | |  | Indirectness | | | |  |
| --- | --- | --- | --- | --- | --- | --- | --- | --- | --- | --- | --- | --- |
| Study name | Study design | Bias arising from the randomization process | Bias due to deviations from intended interventions | Bias due to missing outcome data | Bias in measurement of the outcome | Bias in selection of the reported result | Summary | Population | Intervention | Control | Outcome | Summary |
| SPRINT | RCT | -1 | -1 | 0 | 0 | 0 | -1 | -2 | 0 | 0 | 0 | -2 |
| ALDO-HF | RCT | -1 | 0 | 0 | 0 | 0 | -1 | 0 | -1 | -1 | -1 | -1 |
| PARAMOUNT | RCT | -1 | 0 | 0 | 0 | 0 | -1 | 0 | -1 | -1 | 0 | -1 |
| TOPCAT | RCT | 0 | 0 | 0 | 0 | 0 | 0 | -1 | -1 | -1 | 0 | -1 |
| STTRONG-HF | RCT | 0 | -1 | 0 | 0 | 0 | -1 | -1 | -1 | -1 | -1 | -1 |

HF admission

| STUDY | | Risk of Bias | | | | |  | Indirectness | | | |  |
| --- | --- | --- | --- | --- | --- | --- | --- | --- | --- | --- | --- | --- |
| Study name | Study design | Bias arising from the randomization process | Bias due to deviations from intended interventions | Bias due to missing outcome data | Bias in measurement of the outcome | Bias in selection of the reported result | Summary | Population | Intervention | Control | Outcome | Summary |
| SPRINT | quasi- RCT | -1 | -1 | 0 | 0 | 0 | -1 | -2 | 0 | 0 | 0 | -2 |
| PARAMOUNT | RCT | -1 | 0 | 0 | 0 | 0 | -1 | 0 | -1 | 0 | 0 | -1 |
| TOPCAT | RCT | 0 | 0 | 0 | 0 | 0 | 0 | -1 | -1 | 0 | 0 | -1 |
| STTRONG-HF | RCT | 0 | -1 | 0 | 0 | 0 | 0 | -1 | -1 | 0 | 0 | -1 |
| PARAGON-HF | RCT | -1 | 0 | 0 | 0 | 0 | -1 | -1 | -1 | -1 | 0 | -1 |

Renal dysfunction

| STUDY | | Risk of Bias | | | | |  | Indirectness | | | |  |
| --- | --- | --- | --- | --- | --- | --- | --- | --- | --- | --- | --- | --- |
| Study name | Study design | Bias arising from the randomization process | Bias due to deviations from intended interventions | Bias due to missing outcome data | Bias in measurement of the outcome | Bias in selection of the reported result | Summary | Population | Intervention | Control | Outcome | Summary |
| ALDO-HF | RCT | -1 | 0 | 0 | 0 | 0 | -1 | 0 | -1 | -1 | -1 | -1 |
| PARAMOUNT | RCT | -1 | 0 | 0 | 0 | 0 | -1 | 0 | -1 | -1 | -1 | -1 |
| STTRONG-HF | RCT | 0 | -1 | 0 | 0 | 0 | -1 | -1 | -1 | -1 | -1 | -1 |
| PARAGON-HF | RCT | -1 | 0 | 0 | 0 | 0 | -1 | -1 | -1 | -1 | 0 | -1 |

Hypotension

| STUDY | | Risk of Bias | | | | |  | Indirectness | | | |  |
| --- | --- | --- | --- | --- | --- | --- | --- | --- | --- | --- | --- | --- |
| Study name | Study design | Bias arising from the randomization process | Bias due to deviations from intended interventions | Bias due to missing outcome data | Bias in measurement of the outcome | Bias in selection of the reported result | Summary | Population | Intervention | Control | Outcome | Summary |
| PARAMOUNT | RCT | -1 | 0 | 0 | 0 | 0 | -1 | 0 | -1 | -1 | 0 | -1 |
| STTRONG-HF | RCT | 0 | -1 | 0 | 0 | 0 | -1 | -1 | -1 | -1 | -1 | -1 |
| PARAGON-HF | RCT | -1 | 0 | 0 | 0 | 0 | -1 | -1 | -1 | -1 | -1 | -1 |

SAEs

| STUDY | | Risk of Bias | | | | |  | Indirectness | | | |  |
| --- | --- | --- | --- | --- | --- | --- | --- | --- | --- | --- | --- | --- |
| Study name | Study design | Bias arising from the randomization process | Bias due to deviations from intended interventions | Bias due to missing outcome data | Bias in measurement of the outcome | Bias in selection of the reported result | Summary | Population | Intervention | Control | Outcome | Summary |
| ALDO-HF | RCT | 0 | 0 | 0 | 0 | 0 | 0 | 0 | -1 | -1 | -1 | -1 |
| STTRONG-HF | RCT | 0 | -1 | 0 | 0 | 0 | -1 | -1 | -1 | -1 | 0 | -1 |
| PARAGON-HF | RCT | -1 | 0 | 0 | 0 | 0 | -1 | -1 | -1 | -1 | 0 | -1 |
